# Self-assembled microtissues loaded with osteogenic MSCs for *in vivo* bone regeneration

**DOI:** 10.3389/fbioe.2022.1069804

**Published:** 2022-12-12

**Authors:** Hui Li, Zihao He, Wenjing Li, Jiao Jiao Li, Jianhao Lin, Dan Xing

**Affiliations:** ^1^ Arthritis Clinic and Research Center, Peking University People’s Hospital, Peking University, Beijing, China; ^2^ Arthritis Institute, Peking University, Beijing, China; ^3^ MOE Key Laboratory of Bioorganic Phosphorus Chemistry and Chemical Biology, Department of Biomedical Engineering, School of Medicine, Tsinghua-Peking Center for Life Sciences, Tsinghua University, Beijing, China; ^4^ Kolling Institute, University of Sydney, Sydney, NSW, Australia

**Keywords:** bone regeneration, microtissues, gelatin microcryogel, mesenchymal stem cells, self-assembly

## Abstract

Bone regeneration strategies based on mesenchymal stem cell (MSC) therapy have received widespread attention. Although MSC incorporation into bone scaffolds can help with the repair process, a large number of studies demonstrate variable effects of MSCs with some noting that the inclusion of MSCs does not provide better outcomes compared to unseeded scaffolds. This may in part be related to low cell survival following implantation and/or limited ability to continue with osteogenic differentiation for pre-differentiated cells. In this study, we incorporated MSCs into gelatin microcryogels to form microtissues, and subjected these microtissues to osteogenic induction. We then mixed as-formed microtissues with those subjected to 6 days of osteogenic induction in different ratios, and investigated their ability to induce *in vitro* and *in vivo* osteogenesis during self-assembly. Using a full-thickness rat calvarial defect model, we found that undifferentiated and osteogenically induced microtissues mixed in a ratio of 2:1 produced the best outcomes of bone regeneration. This provides a new, customizable cell-based therapeutic strategy for *in vivo* repair of bone defects.

## Introduction

Between 5% and 20% of all bone fractures result in delayed healing or non-union, with the overall rate of non-union estimated at 1.9%–10%, leading to chronic morbidity, prolonged hospitalization and increased costs (Gómez-Barrena et al., 2015; Zura et al., 2016). The rate of fracture non-union varies depending on the anatomical region. For instance, femoral shaft non-union is estimated to occur in 8% of patients treated with intramedullary nailing (Rupp et al., 2018). The cost of fracture non-union poses a significant healthcare burden, for example, tibial shaft non-union amounts to a median total cost of $25,556 in the United States, more than double the cost compared to tibial shaft fractures that achieve union within 24 months after fracture (Antonova et al., 2013).

Mesenchymal stem cell (MSC)-based bone regeneration strategies have received increasing attention in the past decades. Both autologous (Bhattacharjee et al., 2019) and allogeneic (Arinzeh et al., 2003) MSC therapy can play an important role in bone reconstruction and have been adopted using various approaches. These approaches include MSC cell therapy, MSC secretome therapy (including conditioned medium, extracellular vesicles, and other secretory products), and MSC-loaded carriers (including cell-seeded demineralized bone matrix, bone substitutes, and scaffolds) (Liebergall et al., 2013). Among these, scaffold-based approaches and cell carriers have distinct advantages, as they provide a supportive matrix for MSCs to be implanted into the bone defect area, and can directly participate in promoting local bone reconstruction. The scaffold or cell carrier should ideally mimic the structure and function of the bone extracellular matrix (ECM) (Yu et al., 2015), and provide a functional three-dimensional space for the adhesion, migration, proliferation, and differentiation of osteoblast progenitors (Venkataiah et al., 2021), as well as help to maintain the osteogenic activity of loaded cells following implantation.

Although MSC incorporation into bone scaffolds has been shown to help with bone reconstruction, a large number of studies have also demonstrated variable outcomes of cell-seeded scaffolds, with some noting that the inclusion of MSCs does not necessarily provide better outcomes compared to cell-free scaffolds in preclinical models of bone repair. The beneficial effects of MSCs might be hindered by a range of factors including low cell survival after implantation and limited ability of the scaffold to sustain osteogenic differentiation and continuous bone formation (Shang et al., 2021). These factors can often lead to failure of bone repair particularly in large bone defects (Scarano et al., 2017).

Previous experimental strategies combining MSCs with a cell carrier or scaffold to induce bone regeneration have focused almost exclusively on improving the properties of the supporting matrix to sustain cell survival and osteogenic capacity. However, the potential benefits of modulating the composition of the transplanted cells should not be ignored. During the process of osteoblastic differentiation, various osteogenically inducing factors are produced at different stages including RUNX2, ALP, COL1A1, OPN, and OCN. These factors can help propel the proliferation, collagen expression, mineralization, and other osteogenic processes to sustain ongoing bone formation (Nakashima et al., 2002; Ai-Aql et al., 2008; Vimalraj, 2020). On the other hand, transplanting undifferentiated MSCs makes better use of their paracrine functions that reduce inflammation and improve tissue healing. Current cell-based bone regeneration strategies have generally focused on incorporating either osteogenically primed or undifferentiated stem cells into implantable matrices. In this study, we hypothesize that better osteogenic outcomes can be achieved by mixing populations of undifferentiated and osteogenically primed MSCs, which draws benefits both from shared osteogenic factors in the microenvironment produced by osteogenic cells, and undifferentiated MSCs which provide stronger stemness as well as anti-apoptosis and anti-senescence characteristics.

To deliver the mixed MSC populations in a suitable carrier, we have chosen gelatin carriers due to their good biocompatibility, biodegradability, and demonstrated use in bone tissue engineering (Kuttappan et al., 2016). In our previous studies, we have successfully developed and applied gelatin microcryogels loaded with MSCs in a variety of tissue regeneration applications (Xing et al., 2020). Using this strategy, we can produce self-assembled MSC-containing microtissues which help to maintain MSC secretory activity and pro-regenerative functions both *in vitro* and *in vivo*. In this study, we explore for the first time the effects of mixing microtissues containing undifferentiated MSCs together with microtissues containing osteogenically primed MSCs in bone regeneration. We demonstrate that undifferentiated and osteogenically primed microtissues mixed in different ratios can significantly change *in vivo* bone regeneration outcomes, and coupling the paracrine activity of undifferentiated MSCs with an osteogenic microenvironment provided by osteogenic MSCs is beneficial for bone repair.

## Materials and methods

### Isolation, culture and identification of rat bone marrow-derived MSCs

Bone marrow was collected from the femur of 8 week old healthy Sprague Dawley rats. The extracted bone marrow was cultured in complete growth medium (α-MEM (Hyclone, United States) supplemented with 10% fetal bovine serum (FBS, Gibco, United States) and 1% penicillin/streptomycin (Hyclone, United States)) in a 10 cm cell culture dish. Each dish contained 5 ml bone marrow and the culture medium was replaced every two days. Adherent cells remaining in the dish were identified as bone marrow MSCs, and cells from the third passage were used for subsequent experiments.

To verify the multilineage differentiation ability of isolated rat bone marrow MSCs, cells were cultured in osteogenic, chondrogenic, and adipogenic medium. To evaluate osteogenesis, cells were stained for alkaline phosphatase at 14 days using BCIP/NBT kit (Beyotime, China) according to the manufacturer’s instructions. To evaluate chondrogenesis, pelleted cells were stained with Alcian blue staining solution at 21 days (Solarbio, China) to visualize proteoglycan deposition. To evaluate adipogenesis, cells were stained with Oil Red O at 21 days (Beyotime, China) to visualize lipid droplets.

To identify MSC-related surface markers, 1 × 106 cells were incubated with the following monoclonal antibodies: PE-CD90 (Invitrogen, United States), PE-CD105 (Invitrogen, United States), FITC-CD34 (proteintech, United States), and FITC-CD45 (proteintech, United States). Unstained cells were used as negative controls. Cells incubated with antibodies at 25°C for 30 min, followed by washing with phosphate buffered saline (PBS) and analysis by flow cytometry (BD Biosciences, United States).

### Fabrication of gelatin microcryogels

Gelatin microcryogels were synthesized according to our published methods (Xing et al., 2020). Briefly, poly (methyl methacrylate) (PMMA) stencil array chips were made by a laser fabrication system. Each chip contained 600 circular micro-wells with diameter of 200 μm. Gelatin solution (4% w/v) was dissolved at 65°C for 45 min, followed by ice bath for 5 min, and 0.5% glutaraldehyde solution was used as crosslinking agent. Gelatin solution was pipetted into the PMMA chip, which then underwent cryogelation for 16 h at –20°C, followed by lyophilization for 1 h. The resulting gelatin microcryogels were washed with 0.1 M NaBH4 (Aladdin, China) solution and distilled water. Microcryogels were lyophilised and vacuum-packaged until use for subsequent experiments.

### Preparation and characterization of self-assembled microtissues

**FIGURE 1:**
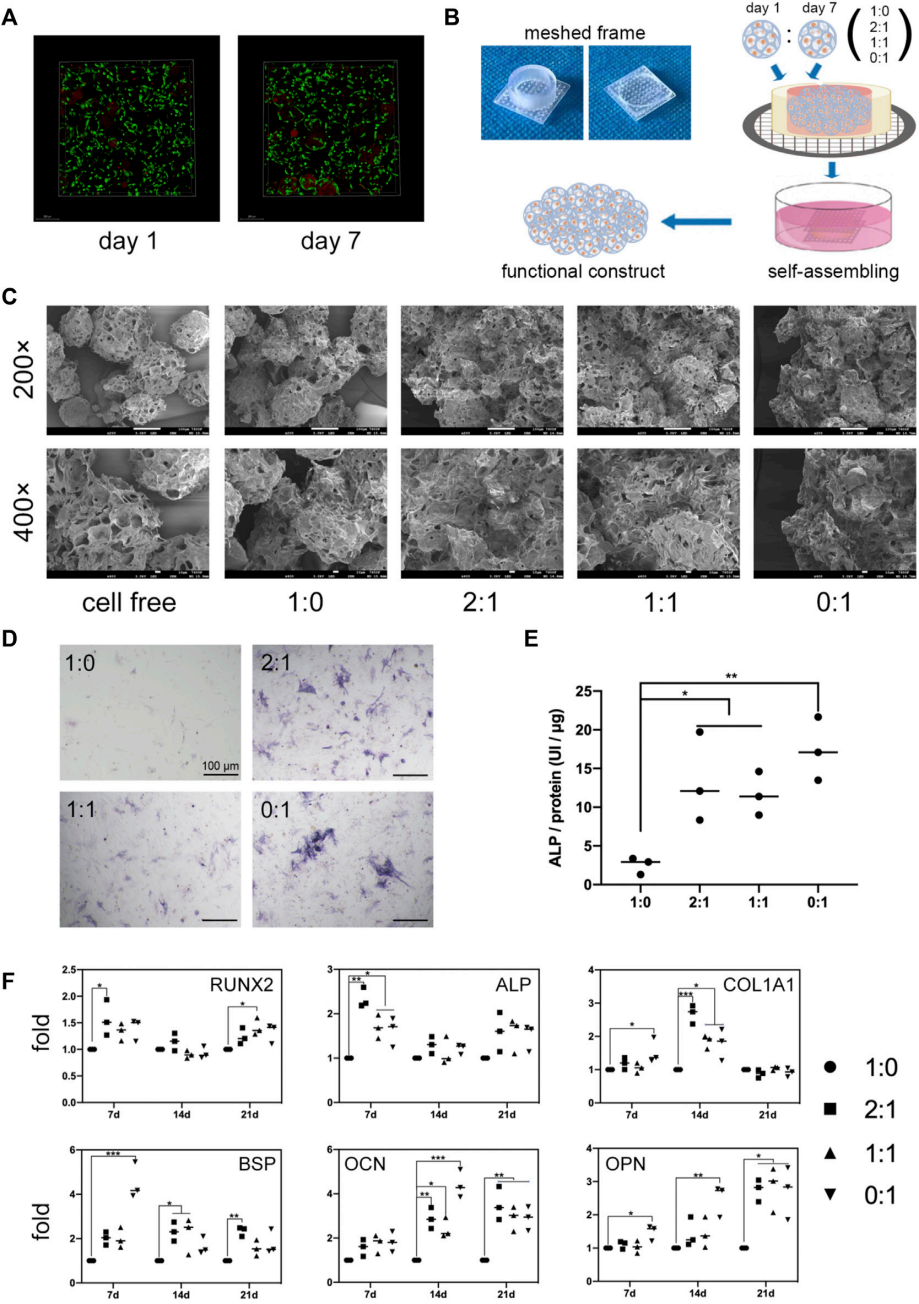
*In vitro* microtissue construct formation and osteogenic differentiation. **(A)**. Live/dead staining of MSCs after 24 h culture in growth medium within microcryogels (day 1) or after additional 6-day osteogenic induction within microcryogels (day 7). **(B)**. Schematic of the process of forming different group of microtissue constructs by *in vitro* self-assembly of MSC-loaded microtissues within meshed frames. **(C)**. Scanning electron microscope (SEM) images of different groups of microtissue constructs cultured in meshed frames for 7 days. **(D)**. ALP staining images of MSCs lysed from different groups of microtissue constructs after 7-day self-assembly. Scale bar = 100 μm. **(E)**. ALP quantitative analysis of different groups of microtissue constructs after 7-day self-assembly (*p < 0.05, **p < 0.01). **(F)**. The mRNA levels of osteogenic differentiation related genes in different groups of microtissue constructs over 21 days (*p < 0.05, **p < 0.01, ***p < 0.001).

Osteogenic medium containing 89% α-MEM, 10% FBS, 1% penicillin/streptomycin, 10 mM β-glycerophosphate, 50 nM ascorbic acid, and 100 nM dexamethasone. Gelatin microcryogels were loaded with MSCs and dispersedly cultured for either 1 day in growth medium (day 1 microtissues in Figure 1A) or additional 6 days in osteogenic medium (day 7 microtissues in Figure 1A). They were then added in different ratios into a meshed frame to induce self-assembly and form microtissue constructs (Figure 1B). Meshed frames were designed using AUTOCAD software and made by 3D printing using poly (lactic acid) (PLA) with 5.5 mm (diameter) × 2.5 mm (height) inner dimensions. The diameter of mesh spacing was set at 100 μm to prevent overflow of microcryogels. The undifferentiated and osteogenically induced MSC-loaded microtissues were mixed in different ratios (1:0, 2:1, 1:1, and 0:1) and cultured in meshed frames within a 6-well plate for 7 days to form microtissue constructs for further analysis.

### Characterization of MSCs in microtissues

The viability of MSCs within microtissues was characterized by live/dead staining (Life Technologies, United States). Morphological observation was performed by scanning electron microscopy (SEM, FEI Quanta 200, Netherlands). Microtissue constructs were completely removed from the meshed frames after 7 days culture, fixed by 2.5% glutaraldehyde, dehydrated by graded ethanol, lyophilised, and sputtered with gold for SEM analysis.

### Characterization of osteogenic differentiation

To evaluate osteogenic differentiation, microtissue constructs grown in meshed frames for 7 days were removed from the frame, and MSCs were digested from the constructs using 0.1% collagenase I for 30 min at 37°C. MSCs were then seeded in 48-well plates and cultured for 8 h in growth medium. An ALP staining kit (Beyotime, China) was used to visualize ALP expression according to the manufacturer’s instructions. Whole microtissue constructs were used to quantitatively analyze ALP protein level, where 0.2% Triton X-100 was used to repeatedly blow the constructs after 7-day self-assembly. After centrifugation, the supernatant was used to quantify ALP levels using an ALP quantitative analysis kit (Beyotime, China) according to the manufacturer’s instructions.

Microtissue constructs cultured in meshed frames for 3, 7, and 14 days were subjected to gene expression analysis for markers of osteogenic differentiation by quantitative real-time PCR (qPCR). The constructs were homogenized in TRIZOL reagent (Life Technologies. United States), and total RNA extraction was performed following the manufacturer’s instructions. cDNA was synthesized from 500 ng of DNA-free total RNA using PrimeScript RT Reagent Kit (Taraka. Japan). Gene-specific transcription was analyzed by qPCR using SYBR Premix Ex Taq II (Taraka. Japan) on a CFX96 instrument (Bio-Rad. United States). All genes were normalized to GAPDH. Primers used are listed in Table 1.

**TABLE 1:** Primer sequences.

	Forward primer	Reverse primer
RUNX2	GGT​GGA​GCT​ACG​GAC​AAT​GAA​TGG	GCT​TGA​GGC​ACT​GAC​TGA​GAC​TG
ALP	CAC​GGC​GTC​CAT​GAG​CAG​AAC	CAG​GCA​CAG​TGG​TCA​AGG​TTG​G
COL1A1	TGT​TGG​TCC​TGC​TGG​CAA​GAA​TG	GTC​ACC​TTG​TTC​GCC​TGT​CTC​AC
BSP	AAG​CGA​CGA​GGA​AGA​GGA​AGA​GG	TTG​GTG​CTG​GTG​CCG​TTG​AC
OCN	GGA​CCC​TCT​CTC​TGC​TCA​CTC​TG	ACC​TTA​CTG​CCC​TCC​TGC​TTG​G
OPN	GAC​GAT​GAT​GAC​GAC​GAC​GAT​GAC	GTG​TGC​TGG​CAG​TGA​AGG​ACT​C

### Characterization of cell apoptosis and senescence

For cell apoptosis analysis, MSCs were digested from microtissue constructs using the same procedures as above. The MSC suspension was stained using annexin V/propidium iodide (PI) double staining apoptosis detection kit (Beyotime, China) according to the manufacturer’s instructions, and analyzed by flow cytometry (BD Fortessa, United States).

The β-galactosidase (β-Gal) activity of MSCs was measured using a senescence β-galactosidase staining kit (Beyotime, China) according to the manufacturer’s instructions. MSCs were digested from microtissue constructs, seeded in a 6-well plate and cultured for 8 h. The MSCs were observed using an inverted microscope (Olympus, Japan), and the number of senescent cells were counted in three randomly selected high-power fields.

### *In vivo* study and analysis of bone regeneration

Sprague-Dawley female rats (8 week old, 220–260 g) were used for *in vivo* evaluation of bone regeneration using microtissues. A full-thickness calvarial defect with a diameter of 5 mm was constructed on the top of the rat skull. Rats were divided into 5 groups (*n* = 6) and implanted with: cell free microcryogels, and microtissues comprising undifferentiated/osteogenically primed MSC-loaded microcryogels in ratios of 1:0, 2:1, 1:1, and 0:1. PLA mesh was removed before implantation. Rats were sacrificed by excessive administration of anesthesia at 3 months after surgery. Explanted calvarial samples containing the defect were fixed in 4% paraformaldehyde for 24 h, and then evaluated by micro-CT (SCANCO μCT-100, Switzerland). The bone volume/tissue volume (BV/TV) and bone mineral density (BMD) of the regenerated bone was measured by Evaluation V6.5 (SCANCO, Switzerland).

For histological analysis, the samples were decalcified in 10% EDTA for 4 weeks. After dehydration by graded ethanol, samples were embedded in paraffin. Decalcified paraffin sections with 7 mm thickness were stained using hematoxylin and eosin (H&E) and Masson’s trichrome.

For mineralization rate analysis, alizarin red (30 mg/kg, Sigma-Aldrich, United States) and calcein (30 mg/kg, Sigma-Aldrich, United States) with fluorescent labeling were injected intraperitoneally at 3 and 21 days before euthanasia, respectively. Sample collection and histological processing were performed as described above. Non-decalcified sections were observed using a fluorescence microscope (Olympius, Japan).

### Statistical analysis

All data were obtained from at least 3 independent experiments, and expressed as mean ± standard deviation. After testing for homogeneity of variances, one-way analysis of variance (ANOVA) followed by Tukey’s multiple comparisons test were used to determine significant differences between groups. *p* < 0.05 was considered a significant difference between groups.

## Results

### *In vitro* microtissue formation and osteogenic differentiation

The trilineage differentiation ability (Supplementary Figure S1A) and surface markers (Supplementary Figure S1B) of isolated rat bone marrow MSCs were verified. Microcryogels loaded with MSCs for 24 h were defined as day 1 microtissues, while day 1 microtissues subjected to osteogenic induction in differentiation medium for additional 6 days were defined as day 7 microtissues. Both day 1 and day 7 microtissues underwent dispersed culture. Live/dead staining showed that MSCs in the day 1 and day 7 microtissue had good cell activity (Figure 1A). Taking advantage of the ability of cell-laden microtissues to self-assemble *in vitro*, we combined day 1 and day 7 microtissues in different proportions (1:0, 2:1, 1:1, and 0:1) to form customized microtissue constructs, and investigated the cell survival and osteogenic differentiation potential of MSCs in these self-assembly constructs (Figure 1B).

We used SEM to characterize the self-assembly of microtissues with different proportions in meshed frames for 7 days. SEM images showed that all groups of microtissues containing MSCs underwent self-assembly, and fusion between adjacent microtissues was achieved through ECM-like substance. Meanwhile, the cell free microcryogels remained separate and were not joined to each other. The degree of self-assembly appeared to be higher for constructs containing day 7 (osteogenic) microtissues (2:1, 1:1, 0:1 groups) compared to constructs containing only day 1 microtissues. This suggests the introduction of an osteogenic microenvironment may promote ECM production by MSCs and lead to better self-assembly.

The state of osteogenic differentiation for different groups of microtissue constructs was measured after 7 days of culture in meshed frames. MSCs extracted from microtissue constructs showed more staining and higher protein levels of ALP for constructs containing day 7 (osteogenic) microtissues (2:1, 1:1, 0:1 groups) compared to the group that contained only day 1 microtissues (Figures 1D,E). qPCR results showed that the same groups containing day 7 (osteogenic) microtissues had significantly higher transcription levels of key osteogenic genes over 21 days, including RUNX2 and ALP indicating early-stage osteogenic differentiation, COL1A1 and BSP indicating middle-stage differentiation, and OCN and OPN indicating later stage differentiation (Figure 1F). Although the constructs containing day 7 (osteogenic) microtissues showed better osteogenic potential compared to the group without, their osteogenic potential was not correlated with the ratio of incorporation of day 7 microtissues within the constructs.

### *In vitro* survival of MSCs within microtissues

**FIGURE 2:**
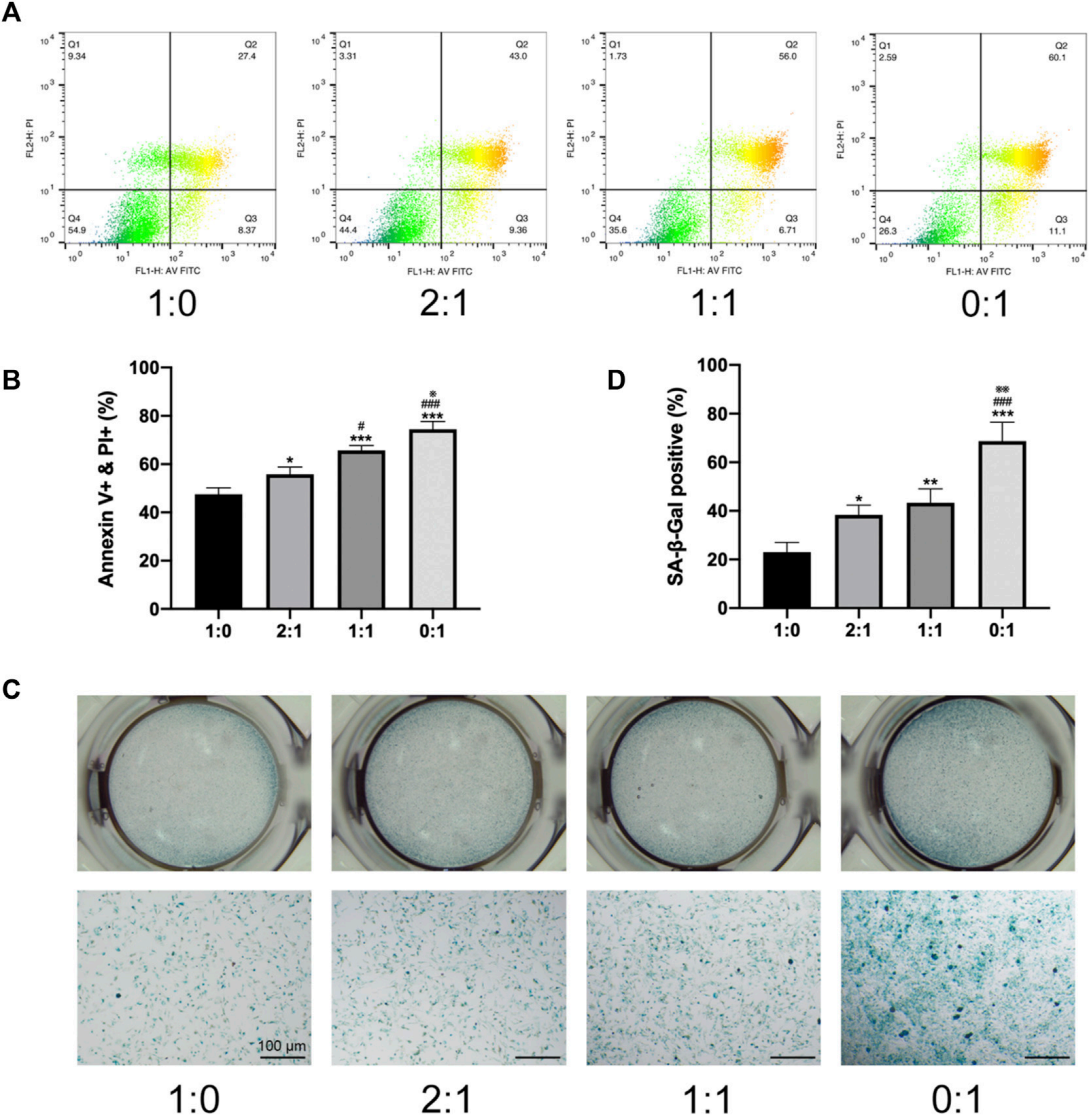
*In vitro* survival of MSCs within microtissue constructs. **(A)**. Apoptosis of MSCs in different groups of microtissue constructs measured by flow cytometry. **(B)**. Quantitative analysis of the proportion of Annexin V positive and PI positive cells. **(C)**. SA-β-Gal staining images of for cells lysed from different groups of microtissue constructs. Scale bar = 100 μm. **(D)**. Quantitative analysis of SA-β-Gal positive cells. (* compared to 1:0 group. # compared to 2:1 group. ※ compared to 1:1 group. */#/※*p* < 0.05, **/##*p* < 0.01, ***/###*p* < 0.001).

To investigate changes in the survival of MSCs in different groups of microtissue constructs during the process of *in vitro* self-assembly, MSC apoptosis was evaluated after 3 days of culturing MSC-loaded microtissues in meshed frames, by flow cytometry (Figure 2A) and quantitative analysis of the proportion of Annexin V and PI positive cells (Figure 2B), as well as SA-β-Gal staining (Figure 2C) and quantitative analysis (Figure 2D). MSCs in non-differentiated constructs containing only day 1 microtissues (1:0 group) showed the lowest level of apoptosis, and the proportion of apoptotic cells progressively increased when higher ratios of day 7 (osteogenic) microtissues were incorporated within the constructs (progressively higher apoptosis for 2:1, 1:1, and 0:1 groups). The different analysis methods showed consistent results on the level of MSC apoptosis in different groups. Constructs that contained both undifferentiated (day 1) and osteogenic (day 7) microtissues were considered to provide the best balance between enhanced osteogenic potential and reduced senescence/apoptosis of MSCs.

### Bone repair using microtissues in rat calvarial bone defects

**FIGURE 3:**
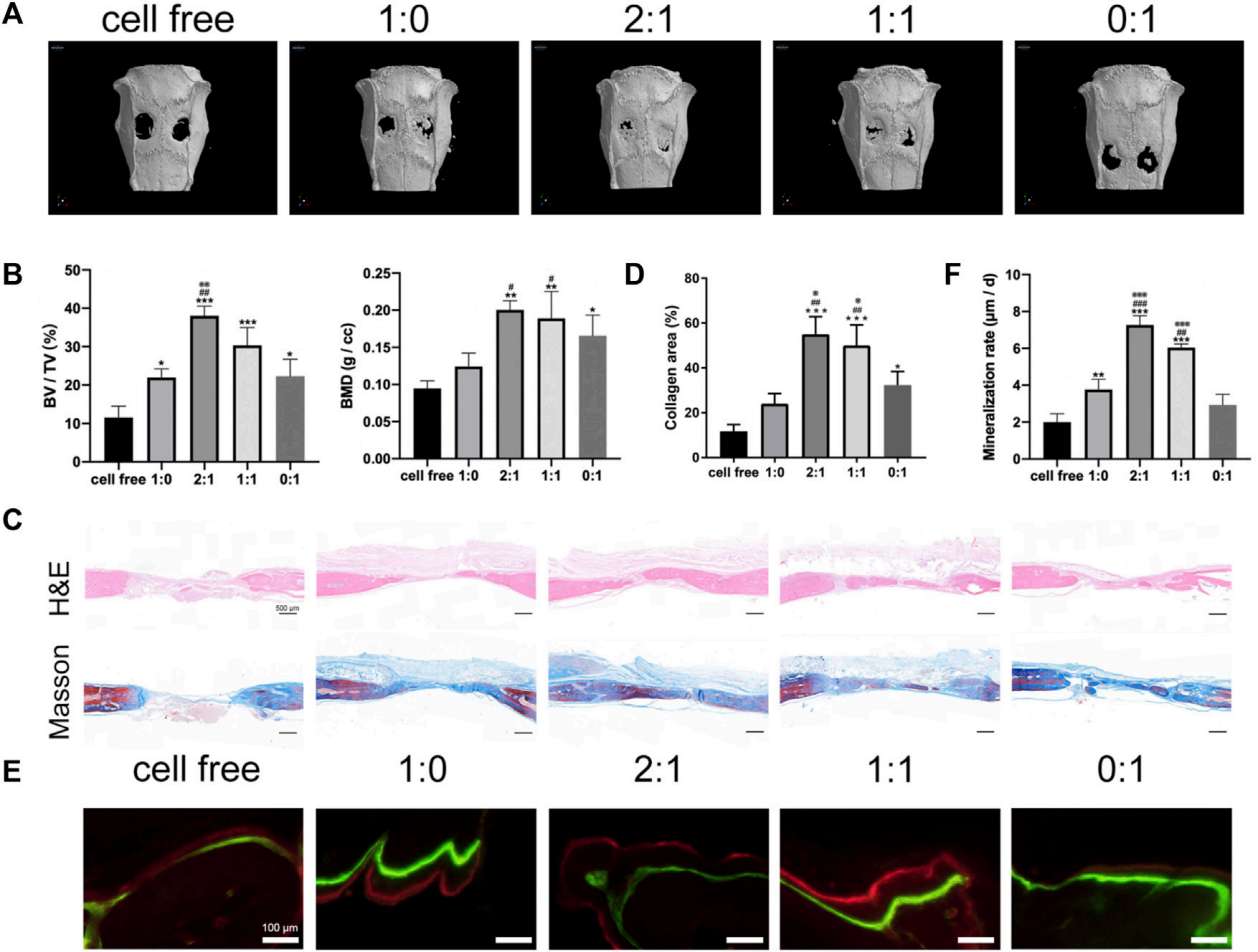
Bone repair using microtissue constructs in rat calvarial bone defects. **(A)**. Micro-CT images of calvarial defect repair effect at 3 months after surgery. **(B)**. Quantitative analysis of new bone volume/tissue volume (BV/TV) and bone mineral density (BMD). **(C)**. Histological analysis by H&E and Masson’s trichrome staining. Scale bar = 500 μm. **(D)**. Quantitative analysis of percentage of collagen in defect area. (* compared to cell free group. # compared to 1:0 group. ※ compared to 0:1 group. */※ *p* < 0.05, ##*p* < 0.01, ***p < 0.001). **(E)**. Tissue mineralization shown by: green = calcein injected 3 weeks before euthanasia, red = alizarin red injected 3 days before euthanasia. Scale bar = 100 μm. **(F)**. Quantitative analysis of mineralization rate (MAR, the average distance between two lines divided by the number of days). (* compared to cell free group. ^#^ compared to 1:0 group. ※ compared to 0:1 group. */^#^ *p* < 0.05, **/^##^/※※ *p* < 0.01, ***/^###^/※※※ *p* < 0.001).

Different groups of microtissue constructs were implanted in a critical-sized rat calvarial defect model, using cell free microcryogels as a control group. The outcomes of bone healing were evaluated by micro-CT (Figures 3A,B), histology (Figures 3C,D), and mineralization (Figures 3E,F) at 3 months after implantation. Micro-CT analysis showed limited bone healing in the cell free control group. New bone formation was most pronounced in the construct groups with a mixed ratio of day 1 and day 7 microtissues (2:1 and 1:1 groups), with macroscopically better defect healing (Figure 3A) and higher BV/TV as well as BMD compared to construct groups containing only day 1 or only day 7 microtissues (Figure 3B). This was further verified by histological staining using H&E and Masson’s trichrome, where continuous bone formation was observed in the defects treated with 2:1 and 1:1 groups, while the 1:0 and 0:1 groups showed irregular and discontinuous new tissue (Figures 3C,D). The amount of mineralization in the newly formed bone area showed similar findings as micro-CT and histology, where the 2:1 and 1:1 groups produced the highest mineralization as seen in images (Figure 3E) and quantitative analysis (Figure 3F) compared to the 1:0 and 0:1 groups. Combining all *in vivo* study results, it appears that the 2:1 group gave the highest level of defect filling with new tissue, while the amount of mineralized tissue formation was highest in the 1:1 group.

## Discussion

This study provided a new approach that could potentially be applied to improve the outcomes of regeneration in large bone defects using MSC-based therapy. By introducing MSCs embedded within self-assembled microtissue constructs, and mixing different proportions of undifferentiated and osteogenically induced MSCs, this approach creates an osteogenic microenvironment in the implanted constructs while still incorporating significant “stemness” or strong regenerative activity of the MSCs. Microtissues with a mixture of undifferentiated and osteogenic MSCs were shown to provide better regenerative characteristics *in vitro* and *in vivo* compared to a homogenous population of MSCs which were all at the same differentiation stage.

The qPCR results of microtissue constructs grown *in vitro* were consistent with a number of expected pathways during different stages of *in vivo* osteogenesis. RUNX2 is a master regulator of osteogenesis and initiates early-stage commitment of undifferentiated MSCs to form pre-osteoblasts (Nakashima et al., 2002), acting as a downstream effector the ERK1/2-RUNX2 pathway (Yuh et al., 2020). Following RUNX2 induction, ALP becomes highly expressed during the early-mid stage of osteogenic differentiation and prepares the osteogenic matrix for mineralization (Vimalraj, 2020). This is followed by the expression of mid-late stage osteogenesis markers including OPN and OCN. Osteopontin encoded by OPN is a highly acidic glycoprotein secreted by differentiating cells, which can bind to collagen type I and help promote osteogenesis (McKee and Nanci, 1996; Wang et al., 2016). Osteocalcin encoded by OCN is a vitamin K dependent non-collagen protein in bone tissue specifically produced by non-proliferative osteoblasts, which acts as a late stage osteogenic marker. Both OPN and OCN are involved in the synthesis of bone matrix and maintenance of bone mineralization (Mizokami et al., 2017). The sequence of this expected sequence of osteogenic marker expression was observed in our qPCR data for microtissues containing osteogenically induced MSCs, indicating that these microtissue constructs have osteogenic potential which was also verified by our *in vivo* data.

During skeletal development, MSCs differentiate into osteoblast progenitor cells which are capable of proliferating before becoming mature osteoblasts (Beeravolu et al., 2016). Therefore, a potential method for encouraging bone regeneration is to use immature osteoblastic precursors (Aino et al., 2014). In our study, we found that microtissue constructs consisting solely of osteogenically induced MSCs tended to undergo mineralization and osteoblastic maturation earlier *in vivo* with limited proliferation potential, and may not be the ideal candidate for regeneration of large bone defects. The higher proportion of senescent cells in these microtissue constructs can also produce undesirable inflammatory and degenerative effects within the microenvironment and further deter bone regeneration (Ambrosi et al., 2021). On the other hand, mixing osteogenically primed MSCs with their undifferentiated counterparts makes maximum use of both an osteogenic microenvironment created by the osteogenic MSCs, as well as the paracrine anti-inflammatory and pro-regenerative functions of the undifferentiated MSCs. In our study, this approach produced the best bone regeneration outcomes and may provide new ideas for developing engineered bone grafts.

Some limitations of our study should be considered when interpreting the results. The mechanical microenvironment plays an important role during *in vivo* osteogenesis (Giannoudis et al., 2007) and is responsible for directing the structure and orientation of load-bearing for the newly formed bone. In this study, we used a calvarial defect model to demonstrate proof-of-concept which was a non-loadbearing model. The outcomes and mechanisms of bone regeneration using microtissue constructs need to be investigated in a load-bearing bone regeneration model to demonstrate greater physiological relevance. In addition, osteoblastic differentiation is a complex and continuous process that is coordinated by multiple factors with tight temporal control at different stages of differentiation (Lee et al., 2018). In our study, we have not conducted specific characterization of the activity of MSCs within the microtissue constructs to elucidate their contribution to the differentiation process and therefore progressive bone formation. Future studies analyzing the secretome, such as markers of inflammation, or transcriptome of MSCs within microtissue constructs undergoing *in vivo* osteogenesis will provide insights into the activation of relevant signaling pathways and reveal the precise mechanisms of improved bone healing using microtissue constructs with mixed undifferentiated and osteogenically primed MSCs. Nevertheless, our study provides a new therapeutic perspective and may help the further development of cell-based therapies for the effective treatment of clinically encountered bone defects.

## Data Availability

The original contributions presented in the study are included in the article/Supplementary Material, further inquiries can be directed to the corresponding authors.
