# Supplementary material for: Self-assembled microtissues loaded with osteogenic MSCs for in vivo bone regeneration
Source: Front Bioeng Biotechnol. 2022 Dec 12;10:1069804. doi: 10.3389/fbioe.2022.1069804 (PMC9790896; doi:10.3389/fbioe.2022.1069804)
Supplement: Supplementary file 1 [file Table1.DOCX]

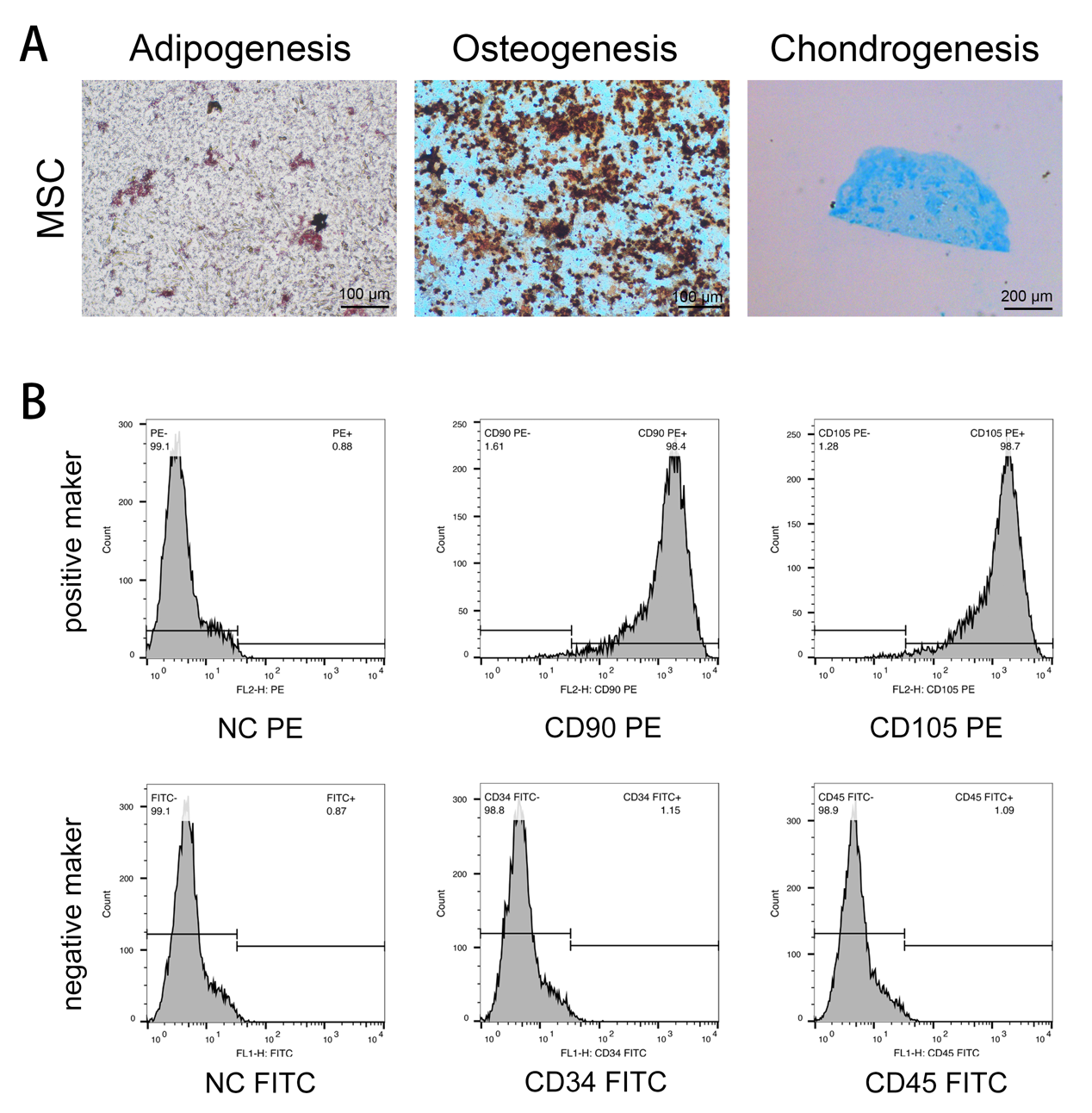


Supplementary Figure 1. A. Trilineage differentiation analysis of rat bone marrow derived MSCs. Scale bar = 100 μm. B. Surface marker analysis of rat bone marrow derived MSCs.
